# Disparities in Influenza Vaccination Coverage and Associated Factors Among Adults with Cardiovascular Disease, United States, 2011–2020

**DOI:** 10.5888/pcd19.220154

**Published:** 2022-10-27

**Authors:** Tarang Parekh, Zulqarnain Javed, Safi U. Khan, Hong Xue, Khurram Nasir

**Affiliations:** 1 Department of Health Administration and Policy, George Mason University, Fairfax, Virginia; 2 Center for Health Data Science and Analytics, Houston Methodist Hospital, Houston, Texas; 3 Houston Methodist DeBakey Heart and Vascular Center, Houston, Texas

## Abstract

**Introduction:**

Influenza vaccination can reduce the incidence of cardiovascular disease (CVD) in the US. However, differences in state-level trends in CVD and sociodemographic and health care characteristics of adults with CVD have not yet been studied.

**Methods:**

In this repeated cross-sectional study, we extracted 476,227 records of adults with a self-reported history of CVD from the Behavioral Risk Factor Surveillance System from January 2011 through December 2020. We calculated the prevalence and likelihood of annual influenza vaccination by sociodemographic characteristics, health care characteristics, and CVD risk factors. Additionally, we examined annual trends of influenza vaccination by geographic location.

**Results:**

The annual age-adjusted influenza vaccination rate among adults with CVD increased from 38.6% (2011) to 44.3% (2020), with an annual average percentage change of 1.1%. Adults who were aged 18 to 44 years, male, non-Hispanic Black/African American, or Hispanic, or had less than a high school diploma, annual household income less than $50,000, and no health insurance had a lower prevalence of vaccination. The odds of vaccination were lower among non-Hispanic Black/African American (adjusted odds ratio, 0.73; 95% CI, 0.70–0.77) and non-Hispanic American Indian/Alaska Native (adjusted odds ratio, 0.86; 95% CI, 0.75–0.98) compared with non-Hispanic White adults. Only 16 states achieved a vaccination rate of 50%; no state achieved the Healthy People 2020 goal of 70%. Nonmedical settings (supermarkets, drug stores) gained popularity (19.2% in 2011 to 28.5% in 2018) as a vaccination setting.

**Conclusion:**

Influenza vaccination among adults with CVD improved marginally during the past decade but is far behind the targeted national goals. Addressing existing disparities requires attention to the role of social determinants of health in determining access to vaccination, particularly among young people, racial and ethnic minority populations, people who lack health insurance, and people with comorbidities.

**Summary**

**What is already known on this topic?**

Influenza vaccination has been shown to reduce cardiovascular illness and death, and routine annual influenza vaccination is recommended by the Centers for Disease Control and Prevention.

**What is added by this report?**

We found marginal improvement in influenza vaccination during the past decade among adults with cardiovascular disease, lagging far behind the Healthy People 2020 goal. Vaccination prevalence is influenced by social determinants of health such as race and ethnicity, access to preventive services, and geographic location.

**What are the implications for public health practice?**

We can achieve Healthy People 2030 goals for vaccine-preventable disease only if we prioritize socially vulnerable populations and look beyond clinical settings as a place of vaccination.

## Introduction

During the past 2 decades, annual influenza vaccination has been a cornerstone of national efforts such as Healthy People to achieve a target vaccination rate of 70% and protect against influenza (1). The American Heart Association and the American College of Cardiology recommend influenza vaccination for secondary prophylaxis of cardiovascular diseases (CVD), reflecting growing evidence of the protective role of vaccination (Class I, Level of Evidence B) (1,2). A recent study reported an increased risk of acute myocardial infarction (AMI) within 7 days of contracting infection with influenza A and influenza B virus (3). Several mechanisms have been proposed to explain the increased risk of CVD, including immune complex deposition in atherosclerotic plaques and subsequent thrombosis and elevated macrophage circulation in arteries (4,5). Current evidence suggests that such adverse outcomes may be prevented with influenza vaccination (3,6,7).

The efficacy of influenza vaccination in preventing AMI has been estimated at 15% to 45%, which is comparable to the documented efficacy of traditional CVD prevention measures such as smoking cessation (32%–43%), statins (19%–30%), and antihypertensive therapy (17%–25%) (6). However, there is a paucity of data on influenza vaccination rates and related sociodemographic differences among adults with CVD. Furthermore, little is known about potential state-level differences in vaccination coverage. To address this gap, we sought to evaluate the national and regional trends of influenza vaccination among adults with CVD. We also examined patterns and predictors of annual influenza vaccination among adults with CVD by key sociodemographic and health care characteristics considered to be social determinants of health.

## Methods

### Data source and study design

We abstracted data from the Behavioral Risk Factor Surveillance System (BRFSS), a nationwide annual telephonic health survey of noninstitutionalized adults aged 18 years or older living in the 50 US states, the District of Columbia, and US territories on health-related risk behaviors, chronic health conditions, and use of preventive services (8). BRFSS is a collaborative project between US states and territories and the Centers for Disease Control and Prevention (CDC). State health departments manage BRFSS field operations with technical assistance from CDC. The structured survey questionnaire is designed and approved by a working group of BRFSS state coordinators and CDC staff members before the beginning of each calendar year. BRFSS conducts surveys via landlines and cellular telephones by using trained survey administrators and random-digit–dialing methods to identify respondents and computer-assisted telephone interview systems to perform structured scripted interviews. For landline telephone sampling, BRFSS divides telephone numbers into 2 strata, high density and medium density, which are determined by the number of listed household numbers in a set of 100 telephone numbers with the same area code, prefix, and first 2 digits of the suffix and all possible combinations of the last 2 digits. For cellular telephone sampling, a commercially available frame is used, whereby the system can call random samples of cellular telephone numbers. The study was determined to be exempt from review by the institutional review board at George Mason University.

We included in our analysis adults aged 18 years or older surveyed from January 2011 through December 2020 with a history of heart attack/myocardial infarction, angina/coronary heart disease (CHD), or stroke. Approximately 6.4% of respondents with CVD were missing information on influenza vaccination and were excluded from our analytic sample. The final sample comprised 476,227 adults with CVD and accounted for 8.5% of the BRFSS survey sample conducted from 2011 through 2020. Median survey responses ranged from 45.1% to 49.9% for the study period.

### Study variables

Annual influenza vaccination was defined as receipt of an influenza vaccination within 12 months before the interview date. Sociodemographic covariates include age (categorized as 18–44, 45–64 years, and ≥65 years), sex (male, female), race and ethnicity (Hispanic, non-Hispanic American Indian/Alaska Native, non-Hispanic Asian, non-Hispanic Black/African American, non-Hispanic Hawaiian/Pacific Islander, non-Hispanic White, and non-Hispanic other), education level (some high school or less, high school graduate, some college or technical school, college graduate), annual household income (<$50,000 or ≥$50,000), marital status (married; unmarried; divorced, widowed, or separated), and US Census–defined geographic region (New England, Middle Atlantic, East North Central, West North Central, South Atlantic, East South Central, West South Central, Mountain, Pacific, US Islands). Health care characteristics were having any health insurance (yes/no), having a personal doctor or health care provider (hereinafter, personal health care provider) (yes/no), and time since the most recent visit to the personal health care provider for a routine checkup. Primary risk factors for CVD included diabetes, obesity (body mass index >30.0), and smoking status (never, former, and current).

### Statistical analysis

The survey procedures (svyset) in Stata version 17.0 (StataCorp LLC) were used to account for the complex sampling design and BRFSS survey weights and to determine national and state-level population estimates. To compute direct age-adjusted estimates, we used 2010 US Census population proportions for groups aged 18 to 44 years, 45 to 64 years, and 65 years or older. We first performed a descriptive analysis of sociodemographic characteristics, health care characteristics, and CVD risk factors, and we used a χ^2^ test to compare the distribution of these characteristics among participants with and without a history of CVD.

For our primary analysis, we examined the age-adjusted frequency distribution (% prevalence and 95% CI) of annual influenza vaccination coverage among adults with CVD each year from 2011 through 2020. We used Joinpoint trend analysis software version 4.9.1.0 (National Cancer Institute) (9) to analyze temporal trends in age-adjusted prevalence of influenza vaccination by years across all characteristics. The Joinpoint regression fits trend data from start to end years and identifies trend segments with significant changes in trend. For each trend segment in the selected model, the annual percentage change (APC) is calculated to characterize trends over time per segment. The average APC (AAPC) for all years (2011–2020) was obtained as a weighted APC. In our trend analysis, with 10 years of data points, the modeling was restricted to a maximum of 2 joinpoints. Modeling selection was based on the permutation test and evaluated if a change occurred in any segment; a *P* value of <.05 was considered significant.

In a secondary analysis, we examined various places for vaccination among participants who reported receiving the vaccine in the past 12 months. BRFSS has the following response options: doctor’s office or health maintenance organization (HMO); health department; another type of clinic or health center (a community health center); senior, recreation, or community center; store (supermarket, drug store); hospital (inpatient); emergency room; workplace; some other kind of place; school; received vaccination in Canada/Mexico; don’t know/not sure; and refused. We combined categories into the following: doctor’s office (including HMO), other health care facility (health department, another type of clinic or health center, and community health center), hospital/emergency room, store, workplace, and other (senior or recreation center, some other kind of place, school, outside US, and don’t know/not sure/refused). This analysis was performed by using the core questionnaire module for the years 2011, 2012, 2015, and 2018. Because of limited years of data for place of vaccination, we did not perform trend analysis and reported only age-adjusted prevalence.

Multivariate logistic regression models were weighted to estimate the adjusted odds ratios (AOR) and 95% CIs of influenza vaccination associated with each sociodemographic characteristic, health care characteristic, and CVD risk factor. Furthermore, to account for possible state-level differences and temporal trends in vaccination rates, we generated year and state fixed-effects logistic regression models. A 2-sided *P* value of .05 was used to determine significance.

## Results

Adults with CVD were more likely than adults without CVD to be aged 65 years or older (51.2% vs 16.9%), male (55.4% vs 47.8%), non-Hispanic White (71.5% vs 64.3%), and a high school graduate or less (52.0% vs 40.3%), and have an annual household income of less than $50,000 (69.4% vs 50.4%) (Supplemental Table 1 in Appendix). The prevalence of diabetes (31.7% vs 9.7%), obesity (38.0% vs 28.8%), and current smoking (20.4% vs 16.5%) was greater among adults with CVD than among adults without CVD. Most adults with CVD had health insurance (91.8%), had a personal health care provider (91.0%), and had a visit with the personal health care provider within the past year (85.8%); the prevalence of each of these characteristics was higher among adults with CVD than among adults without CVD (85.7%, 76.6%, and 69.7%, respectively). The influenza vaccination rate was consistently higher among adults with CVD than among adults without CVD (Supplemental Figure in Appendix); however, the gap in prevalence decreased from 2011 through 2020.

Among adults with CVD, the age-adjusted prevalence of influenza vaccination increased from 38.6% in 2011 to 44.3% in 2020 (Supplemental Table 2 in Appendix) with an average APC of 1.1% (Table 1). The APC in influenza vaccination changed from a 4.5% decrease per year during 2015 through 2018 to a 14.1% increase per year during 2018 through 2020. By type of CVD, vaccination rates were highest among adults with a history of angina/CHD (46.9%) and lowest among adults with a history of myocardial infarction (40.1%) in 2020. Influenza vaccination rates were consistently lower among adults aged 18 to 44 years (vs adults aged 45–64 and ≥65 years) and men (vs women). Among racial and ethnic minority groups in 2020, Asian adults had the highest vaccination rate (50.4%), while American Indian/Alaska Native (40.3%), non-Hispanic Black/African American (43.3%), and Hispanic (36.8%) adults had lower rates.

**Table 1:** Age-Adjusted Prevalence of Influenza Vaccination and Annual Percentage Change by Selected Characteristics, US Adults With Cardiovascular Disease, Behavioral Risk Factor Surveillance System, January 2011–December 2020^a^

Characteristic	Age-adjusted prevalence, %		Average annual percentage change (95% CI)	Annual percentage change (95% CI)		
	2011	2020	2011–2020	Trend segment 1, 2011–2015	Trend segment 2, 2015–2018	Trend segment 3, 2018–2020
**Cardiovascular disease**						
Any cardiovascular disease^a^	38.6	44.3	1.1 (1.1 to 2.6)^b^	1.0 (−0.1 to 2.1)	−4.5 (−8.0 to −0.9)^b^	14.1 (10.0 to 18.4)^b^
Angina/coronary heart disease only	39.1	46.9	2.5 (1.0 to 5.0)^b^	0.6 (−3.5 to 4.9)	−1.4 (−15.2 to 14.7)	10.6 (4.5 to 25.7)^b^
Stroke only	38.6	42.9	1.8 (−3.0 to 6.8)	1.3 (−6.2 to 9.3)	−3.9 (−25.2 to 23.6)	11.9 (−12.4 to 42.8)
Myocardial infarction only	32.8	40.1	2.3 (−6.4 to 11.7)	2.3 (−11.0 to 17.6)	−6.4 (−39.9 to 46.0)	16.7 (−28.8 to 91.1)
≥2 Cardiovascular diseases	43.5	46.6	1.4 (−4.0 to 7.0)	−0.1 (−7.6 to 8.0)	−6.9 (−30.1 to 24.0)	18.5 (−9.7 to 55.5)
**Age, y**						
18–44	27.5	33.3	2.7 (−1.6 to 7.3)	2.2 (−4.6 to 9.5)	−5.6 (−24.6 to 18.1)	17.9 (−5.2 to 46.8)
45–64	42.7	48.1	1.4 (−2.8 to 5.7)	0.4 (−5.5 to 6.6)	−4.3 (−22.7 to 18.6)	12.6 (16.5 to 41.5)^b^
≥65	61.4	67.5	1.2 (−1.5 to 4.0)	0.2 (−3.6 to 4.1)	−2.5 (−15.3 to 12.2)	9.6 (−5.3 to 26.7)
**Sex**						
Male	36.4	41.8	1.6 (1.1 to 2.3)^b^	1.3 (0.2 to 2.3)^b^	−5.1 (−8.3 to −1.9)^b^	13.6 (10.0 to 17.3)^b^
Female	41.1	47.2	2.0 (0.1 to 3.9)^b^	0.6 (−2.2 to 3.4)	−3.8 (−12.6 to 5.9)	14.3 (3.8 to 25.9)^b^
**Race and ethnicity**						
American Indian/Alaska Native, non-Hispanic	41.4	40.3	0.8 (−1.2 to 2.1)	−3.3 (−6.3 to 0.3)	−10.4 (−27.8 to 6.2)	20.9 (−17.6 to 58.1)
Asian, non-Hispanic	34.6	50.4	4.2 (−0.2 to 5.6)	0.9 (−0.5 to 2.3)	−10.2 (−24.1 to 3.6)	1.4 (−31.2 to 49.5)
Black/African American, non-Hispanic	36.2	43.3	3.2 (−5.3 to 12.4)	3.4 (−9.3 to 16.1)	−5.4 (−15.1 to 4.2)	17.0 (−29.6 to 94.6)
Hispanic	33.9	36.8	1.0 (−1.0 to 3.1)	1.2 (−2.1 to 4.6)	−2.5 (−12.0 to 8.0)	6.4 (−4.9 to 19.0)
Native Hawaiian/Pacific Islander, non-Hispanic	39.7	46.1	2.4 (−4.9 to 10.1)	−6.6 (−16.5 to 4.5)	−9.2 (−27.0 to 8.6)	11.5 (−20.0 to 55.4)
White, non-Hispanic	39.7	46.3	1.9 (1.2 to 2.6)^b^	1.1 (−0.1 to 2.2)	−6.1 (−9.6 to −2.5)^b^	17.1 (13.1 to 21.2)^b^
Other, non-Hispanic	36.7	40.4	0.9 (−1.3 to 2.1)	6.8 (−6.7 to 20.1)	−6.0 (−17.1 to 5.0)	3.3 (−24.4 to 41.1)
**Education**						
Some high school or less	32.5	36.2	0.9 (−1.3 to 3.1)	1.9 (−1.5 to 5.5)	−6.1 (−16.2 to 5.3)	9.9 (−2.0 to 23.2)
High school graduate	37.5	41.0	1.1 (−1.9 to 4.1)	−0.9 (−5.3 to 3.6)	−3.0 (−17.0 to 13.3)	11.9 (−4.3 to 30.8)
Some college or technical school	40.4	44.2	1.6 (−0.2 to 3.5)	0.8 (−1.9 to 3.5)	−4.0 (−12.8 to 5.7)	12.6 (3.1 to 23.0)^b^
College graduate	46.2	59.1	2.9 (1.0 to 4.9)^b^	2.3 (−0.9 to 5.6)	−5.4 (−14.1 to 4.3)	18.1 (6.2 to 31.3)^b^
**Annual household income, $**						
<50,000	36.7	41.8	1.7 (−1.1 to 4.6)	0.6 (−3.6 to 5.0)	−4.8 (−17.6 to 10.0)	14.7 (−0.8 to 32.6)
≥50,000	44.5	49.7	1.9 (−3.5 to 7.5)	2.4 (−5.6 to 11.2)	−6.7 (−30.1 to 24.6)	14.8 (−12.3 to 50.4)
**Marital status**						
Married	39.9	50.0	2.5 (1.8 to 3.3)^b^	1.5 (0.4 to 2.5)^b^	−6.2 (−9.8 to −2.6)^b^	19.6 (15.3 to 24.2)^b^
Unmarried	37.8	40.1	1.3 (−3.6 to 6.4)	−1.5 (−15.8 to 15.3)	−0.6 (−14.5 to 15.6)	9.5 (−19.1 to 48.2)
Divorced/widowed/separated	36.6	38.3	1.1 (−0.9 to 3.1)	2.9 (−0.2 to 6.1)	−6.1 (−15.3 to 4.0)	9.1 (−1.5 to 20.9)
**Health insurance**						
No	22.7	24.5	1.6 (−5.4 to 9.1)	0.7 (−10.0 to 12.6)	−4.2 (−33.6 to 38.1)	12.8 (−23.0 to 65.2)
Yes	43.0	48.2	1.5 (−0.7 to 3.8)	0.1 (−3.3 to 3.4)	−4.7 (−15.1 to 7.1)	15.2 (2.9 to 28.9)^b^
**Diabetes**						
No	36.6	42.0	1.9 (0.8 to 3.0)^b^	0.9 (−0.7 to 2.6)	−4.4 (−9.5 to 1.0)	14.2 (8.2 to 20.6)^b^
Yes	46.2	52.1	1.7 (−0.4 to 4.0)	1.8 (−1.7 to 5.3)	−6.4 (−16.6 to 5.0)	15.2 (3.5 to 28.3)^b^
**Obesity (body mass index >30.0)**						
No	38.0	42.3	1.8 (−1.1 to 4.8)	0.5 (−3.7 to 4.9)	−3.7 (−17.1 to 11.9)	13.6 (−2.1 to 31.8)
Yes	40.2	46.6	1.8 (−2.9 to 6.8)	1.4 (−5.9 to 9.2)	−5.0 (−25.6 to 21.3)	13.9 (−11.6 to 46.7)
**Cigarette use**						
Never	41.1	45.9	1.9 (0.1 to 3.8)^b^	0.3 (−2.7 to 3.5)	−3.6 (−12.7 to 6.4)	14.1 (4.1 to 25.1)^b^
Former	41.5	48.7	1.9 (0.6 to 3.2)^b^	1.2 (−0.8 to 3.2)	−5.2 (−10.9 to 0.8)	15.1 (7.5 to 23.2)^b^
Current	32.1	35.9	1.4 (−5.2 to 8.4)	1.7 (−7.2 to 11.5)	−6.2 (−34.2 to 33.6)	13.2 (−20.3 to 60.8)
**Has a personal health care provider**						
No	23.5	26.1	1.8 (−2.4 to 6.2)	0.3 (−6.3 to 7.5)	−6.6 (−25.0 to 16.5)	19.2 (−3.4 to 47.0)
Yes	42.1	47.9	1.8 (0.1 to 3.4)^b^	1.0 (−1.5 to 3.5)	−4.2 (−12.0 to 4.3)	13.2 (4.1 to 23.1)^b^
**Time since most recent visit to personal health care provider for routine checkup**						
Within last year	42.7	48.8	1.7 (0.2 to 3.2)^b^	1.1 (−1.1 to 3.4)	−5.0 (−12.1 to 2.7)	14.1 (6.0 to 22.7)^b^
1–2 Years since last visit	30.7	29.4	−0.1 (−10.2 to 11.1)	1.9 (−13.7 to 20.3)	−8.6 (−4.9 to 17.2)	9.5 (−7.0 to 27.1)
>2 Years since last visit	25.8	20.1	−2.3 (−6.5 to 2.2)	0.4 (−5.8 to 7.0)	−11.9 (−28.3 to 8.3)	8.2 (−19.0 to 34.5)

a Defined as a history of stroke, myocardial infarction, coronary heart disease, or angina. Unweighted total number of cases of cardiovascular disease is 476,227.

b Significant at *P* < .05; determined by permutation test for joinpoint regression.

Although the AAPC in influenza vaccination prevalence among adults aged 45 to 64 years with CVD was a nonsignificant 1.4%, the prevalence increased significantly during 2018 through 2020 (APC, 12.6%) (Table 1). The overall prevalence of influenza vaccination increased among both men and women, with a greater increase during the last trend segment (2018–2020). The AAPC was 2.9% among college graduates, with prevalence ranging from 46.2% in 2011 to 59.1% in 2020. Although the prevalence of influenza vaccination was higher among adults with diabetes than among adults without diabetes, the prevalence increased significantly among adults without diabetes during 2011 through 2020 (AAPC, 1.9%). Among never and former smokers, influenza vaccination increased at a significant AAPC of 1.9%, with a greater increase during 2018 through 2020 among never smokers (APC, 14.1%) and former smokers (APC, 15.1%). Adults with a personal health care provider had consistently higher vaccination rates than adults without one (42.1% vs 23.5% in 2011; 47.9% vs 26.1% in 2020); the AAPC was 1.8% for both groups, and the largest increase for both groups was during 2018 through 2020 (has a personal health care provider, 13.2%; does not have a personal health care provider, 19.2%). The AAPC in the prevalence of influenza vaccination was 1.7% (42.7% in 2011 to 48.8% in 2020) among adults with a visit to a personal health care provider within the past 1 year, −0.1% (30.7% in 2011 to 29.4% in 2020) among adults reporting 1 or 2 years since their most recent visit, and −2.3% (25.8% in 2011 to 20.1% in 2020) among adults reporting more than 2 years since their most recent visit.

**Figure 1:**
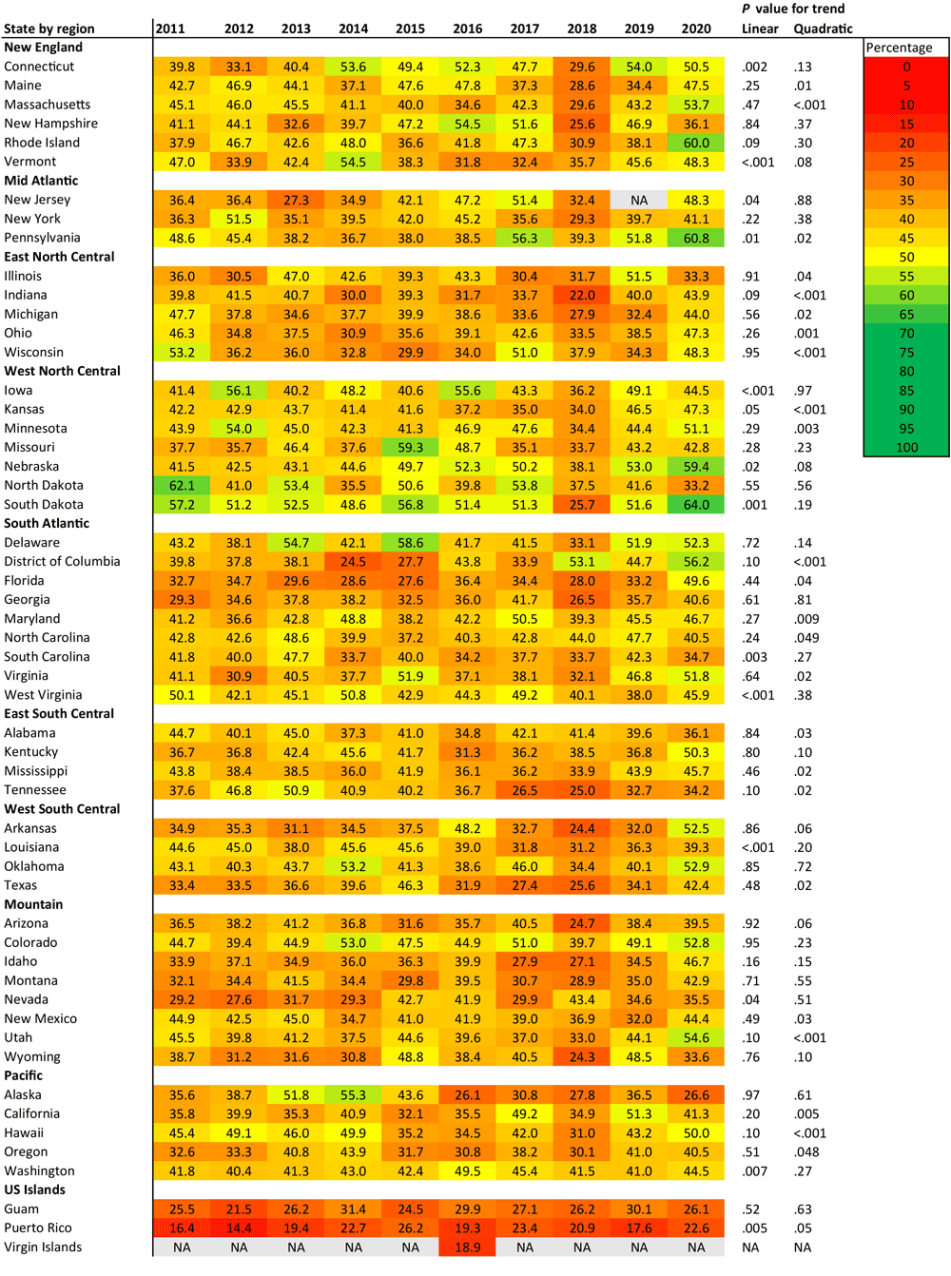
State-specific trends in the prevalence of influenza vaccination among US adults with cardiovascular disease, Behavioral Risk Factor Surveillance System, 2011–2020. Linear and quadratic trends were calculated by using adjusted regression models with survey years modeled as orthogonal polynomials. Abbreviation: NA, not available.

In 2020, the age-adjusted influenza vaccination rate among adults with CVD ranged from 22.6% in Puerto Rico to 64.0% in South Dakota (Figure 1). From 2011 to 2020, the vaccination rate showed a significant positive linear trend in 9 states (Connecticut, Iowa, Nebraska, Nevada, New Jersey, Pennsylvania, South Dakota, Vermont, Washington) and Puerto Rico. A negative linear trend was observed in 3 states (Louisiana, South Carolina, West Virginia). Overall, West North Central region states performed well in influenza vaccination rates during the study period. Only 16 states achieved a vaccination rate of 50%, and no state achieved the Healthy People 2020 goal of 70%.

**Table:** 

State by region	Prevalence										*P* for linear trend	*P* for quadratic trend
	2011	2012	2013	2014	2015	2016	2017	2018	2019	2020		
**New England**												
Connecticut	39.8	33.1	40.4	53.6	49.4	52.3	47.7	29.6	54.0	50.5	.002	.13
Maine	42.7	46.9	44.1	37.1	47.6	47.8	37.3	28.6	34.4	47.5	.25	.01
Massachusetts	45.1	46.0	45.5	41.1	40.0	34.6	42.3	29.6	43.2	53.7	.47	<.001
New Hampshire	41.1	44.1	32.6	39.7	47.2	54.5	51.6	25.6	46.9	36.1	.84	.37
Rhode Island	37.9	46.7	42.6	48.0	36.6	41.8	47.3	30.9	38.1	60.0	.09	.30
Vermont	47.0	33.9	42.4	54.5	38.3	31.8	32.4	35.7	45.6	48.3	.001	.08
**Middle Atlantic**												
New Jersey	36.4	36.4	27.3	34.9	42.1	47.2	51.4	32.4	NA	48.3	.04	.88
New York	36.3	51.5	35.1	39.5	42.0	45.2	35.6	29.3	39.7	41.1	.22	.38
Pennsylvania	48.6	45.4	38.2	36.7	38.0	38.5	56.3	39.3	51.8	60.8	.01	.02
**East North Central**												
Illinois	36.0	30.5	47.0	42.6	39.3	43.3	30.4	31.7	51.5	33.3	.91	.04
Indiana	39.8	41.5	40.7	30.0	39.3	31.7	33.7	22.0	40.0	43.9	.09	<.001
Michigan	47.7	37.8	34.6	37.7	39.9	38.6	33.6	27.9	32.4	44.0	.56	.02
Ohio	46.3	34.8	37.5	30.9	35.6	39.1	42.6	33.5	38.5	47.3	.26	.001
Wisconsin	53.2	36.2	36.0	32.8	29.9	34.0	51.0	37.9	34.3	48.3	.95	<.001
**West North Central**												
Iowa	41.4	56.1	40.2	48.2	40.6	55.6	43.3	36.2	49.1	44.5	<.001	.97
Kansas	42.2	42.9	43.7	41.4	41.6	37.2	35.0	34.0	46.5	47.3	.05	<.001
Minnesota	43.9	54.0	45.0	42.3	41.3	46.9	47.6	34.4	44.4	51.1	.29	.003
Missouri	37.7	35.7	46.4	37.6	59.3	48.7	35.1	33.7	43.2	42.8	.28	.23
Nebraska	41.5	42.5	43.1	44.6	49.7	52.3	50.2	38.1	53.0	59.4	.02	.08
North Dakota	62.1	41.0	53.4	35.5	50.6	39.8	53.8	37.5	41.6	33.2	.55	.56
South Dakota	57.2	51.2	52.5	48.6	56.8	51.4	51.3	25.7	51.6	64.0	.001	.19
**South Atlantic**												
Delaware	43.2	38.1	54.7	42.1	58.6	41.7	41.5	33.1	51.9	52.3	.72	.14
District of Columbia	39.8	37.8	38.1	24.5	27.7	43.8	33.9	53.1	44.7	56.2	.10	<.001
Florida	32.7	34.7	29.6	28.6	27.6	36.4	34.4	28.0	33.2	49.6	.44	.04
Georgia	29.3	34.6	37.8	38.2	32.5	36.0	41.7	26.5	35.7	40.6	.61	.81
Maryland	41.2	36.6	42.8	48.8	38.2	42.2	50.5	39.3	45.5	46.7	.27	.009
North Carolina	42.8	42.6	48.6	39.9	37.2	40.3	42.8	44.0	47.7	40.5	.24	.049
South Carolina	41.8	40.0	47.7	33.7	40.0	34.2	37.7	33.7	42.3	34.7	.003	.27
Virginia	41.1	30.9	40.5	37.7	51.9	37.1	38.1	32.1	46.8	51.8	.64	.02
West Virginia	50.1	42.1	45.1	50.8	42.9	44.3	49.2	40.1	38.0	45.9	<.001	.38
**East South Central**												
Alabama	44.7	40.1	45.0	37.3	41.0	34.8	42.1	41.4	39.6	36.1	.84	.03
Kentucky	36.7	36.8	42.4	45.6	41.7	31.3	36.2	38.5	36.8	50.3	.80	.10
Mississippi	43.8	38.4	38.5	36.0	41.9	36.1	36.2	33.9	43.9	45.7	.46	.02
Tennessee	37.6	46.8	50.9	40.9	40.2	36.7	26.5	25.0	32.7	34.2	.10	.02
**West South Central**												
Arkansas	34.9	35.3	31.1	34.5	37.5	48.2	32.7	24.4	32.0	52.5	.86	.06
Louisiana	44.6	45.0	38.0	45.6	45.6	39.0	31.8	31.2	36.3	39.3	<.001	.20
Oklahoma	43.1	40.3	43.7	53.2	41.3	38.6	46.0	34.4	40.1	52.9	.85	.72
Texas	33.4	33.5	36.6	39.6	46.3	31.9	27.4	25.6	34.1	42.4	.48	.02
**Mountain**												
Arizona	36.5	38.2	41.2	36.8	31.6	35.7	40.5	24.7	38.4	39.5	.92	.06
Colorado	44.7	39.4	44.9	53.0	47.5	44.9	51.0	39.7	49.1	52.8	.95	.23
Idaho	33.9	37.1	34.9	36.0	36.3	39.9	27.9	27.1	34.5	46.7	.16	.15
Montana	32.1	34.4	41.5	34.4	29.8	39.5	30.7	28.9	35.0	42.9	.71	.55
Nevada	29.2	27.6	31.7	29.3	42.7	41.9	29.9	43.4	34.6	35.5	.04	.51
New Mexico	44.9	42.5	45.0	34.7	41.0	41.9	39.0	36.9	32.0	44.4	.49	.03
Utah	45.5	39.8	41.2	37.5	44.6	39.6	37.0	33.0	44.1	54.6	.10	<.001
Wyoming	38.7	31.2	31.6	30.8	48.8	38.4	40.5	24.3	48.5	33.6	.76	.098
**Pacific**												
Alaska	35.6	38.7	51.8	55.3	43.6	26.1	30.8	27.8	36.5	26.6	.97	.61
California	35.8	39.9	35.3	40.9	32.1	35.5	49.2	34.9	51.3	41.3	.197	.01
Hawaii	45.4	49.1	46.0	49.9	35.2	34.5	42.0	31.0	43.2	50.0	.10	<.001
Oregon	32.6	33.3	40.8	43.9	31.7	30.8	38.2	30.1	41.0	40.5	.51	.048
Washington	41.8	40.4	41.3	43.0	42.4	49.5	45.4	41.5	41.0	44.5	.007	.27
**US Islands**												
Guam	25.5	21.5	26.2	31.4	24.5	29.9	27.1	26.2	30.1	26.1	.52	.63
Puerto Rico	16.4	14.4	19.4	22.7	26.2	19.3	23.4	20.9	17.6	22.6	.005	.05
Virgin Islands	NA	NA	NA	NA	NA	18.9	NA	NA	NA	NA	NA	NA

**Figure 2:**
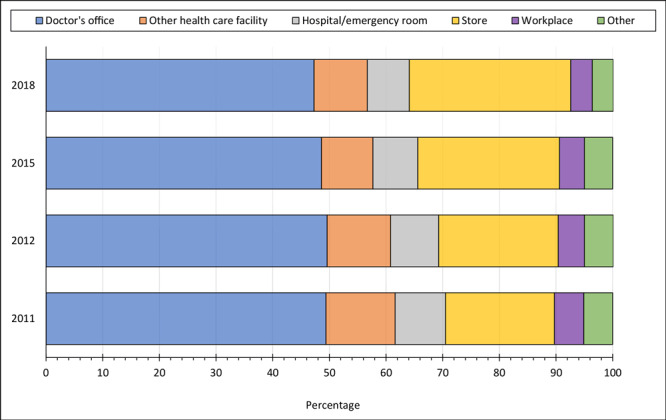
Common places for receiving an annual influenza vaccination among US adults with cardiovascular disease, Behavioral Risk Factor Surveillance System, 2011–2020. “Other health care facility” includes health department, another type of clinic or health center, and a community health center. Store includes supermarkets or drug stores. “Other place” includes senior or recreation center, some other kind of place, school, received outside US, and don’t know/not sure/refused.

Doctors’ offices remained the most common place for annual influenza vaccination among US adults with CVD, despite consistently declining vaccination rates from 2011 (49.4%) to 2018 (47.3%); we observed similar declines for other health care facilities. In contrast, the preference for stores such as supermarkets or drug stores as vaccination sites steadily increased from 19.2% in 2011 to 28.5% in 2018 (Figure 2).

**Table:** 

Place	2011	2012	2015	2018
Doctor’s office	49.4	49.6	48.6	47.3
Other health care facility	12.2	11.2	9.1	9.4
Hospital/emergency room	8.9	8.5	7.9	7.4
Store	19.2	21.1	25	28.5
Workplace	5.2	4.6	4.4	3.8
Other	5.1	5.1	5.0	3.7

Compared with adults with CVD aged 18 to 44 years, adults aged 45 to 64 years (AOR, 1.50; 95% CI, 1.41–1.61) and adults aged 65 years or older (AOR, 2.58; 95% CI, 2.40–2.76) had greater odds of getting an influenza vaccination (Table 2). Women had marginally higher odds (AOR, 1.06; 95% CI, 1.03–1.10) of getting the influenza vaccination than men. Compared with non-Hispanic White adults with CVD, Hispanic adults with CVD had 23% lower odds of getting the annual influenza vaccination (AOR, 0.77; 95% CI, 0.72–0.82) with year-fixed effects, which was not significant when state effects were added. Odds of getting an influenza vaccination were 27% and 14% lower, respectively, among non-Hispanic Black/African American adults (AOR, 0.73; 95% CI, 0.70–0.77) and American Indian/Alaska Native (AOR, 0.86; 95% CI, 0.75- 0.98) adults with CVD compared with non-Hispanic White adults with CVD. The odds of getting an influenza vaccination increased consistently as level of education increased. Adults with CVD and diabetes were 29% more likely to get an influenza vaccination (AOR, 1.29; 95% CI, 1.25–1.33) than adults with CVD and no diabetes. Compared with nonsmoking adults with CVD, former smokers with CVD were 15% more likely to get an influenza vaccination (AOR, 1.15; 95% CI, 1.11–1.19). In contrast, current smokers with CVD were 21% less likely to get an annual influenza vaccination (AOR, 0.79; 95% CI, 0.76–0.83) than nonsmoking adults with CVD. Having health insurance (AOR, 1.76; 95% CI, 1.63–1.89) and a personal health care provider (AOR, 1.71; 95% CI, 1.60–1.83) increased the likelihood of influenza vaccination. The odds of getting an annual influenza vaccination decreased as time increased since the most recent visit to a personal health care provider for a routine checkup.

**Table 2:** Predictors of Influenza Vaccination Among US Adults With Cardiovascular Disease, Behavioral Risk Factor Surveillance System, January 2011–December 2020^a^

Characteristic	Pooled model	Year fixed-effects model^b^	Year–state fixed-effects model^c^
**Age, y**			
18–44	1 [Reference]	1 [Reference]	1 [Reference]
45–64	1.49^d^ (1.40–1.60)	1.49^d^ (1.39–1.59)	1.50^d^ (1.41–1.61)
≥65	2.54^d^ (2.37–2.73)	2.53^d^ (2.36–2.71)	2.58^d^ (2.40–2.76)
**Sex**			
Female	1.06^d^ (1.03–1.09)	1.06^d^ (1.03–1.09)	1.06^d^ (1.03–1.10)
Male	1 [Reference]	1 [Reference]	1 [Reference]
**Race**			
American Indian/Alaska Native, non-Hispanic	0.87^e^ (0.77–1.00)	0.87^e^ (0.76–0.99)	0.86^e^ (0.75–0.98)
Asian, non-Hispanic	1.14 (0.93–1.41)	1.17 (0.96–1.44)	1.21 (0.98–1.50)
Black/African American, non-Hispanic	0.73^d^ (0.70–0.77)	0.73^d^ (0.69–0.77)	0.73^d^ (0.70–0.77)
Hispanic	0.77^d^ (0.72–0.82)	0.77^d^ (0.72–0.82)	0.96 (0.88–1.04)
Native Hawaiian/Pacific Islander, non-Hispanic	0.93 (0.75–1.14)	0.90 (0.73–1.11)	0.91 (0.73–1.12)
White, non-Hispanic	1 [Reference]	1 [Reference]	1 [Reference]
Other, non-Hispanic	0.84^d^ (0.77–0.92)	0.84^d^ (0.76–0.92)	0.84^d^ (0.76–0.92)
**Education**			
Some high school or less	1 [Reference]	1 [Reference]	1 [Reference]
High school graduate	1.07^f^ (1.03–1.13)	1.07^f^ (1.02–1.13)	1.08^f^ (1.03–1.13)
Some college or technical school	1.16^d^ (1.10–1.22)	1.16^d^ (1.11–1.22)	1.18^d^ (1.12–1.24)
College graduate	1.38^d^ (1.31–1.46)	1.38^d^ (1.31–1.46)	1.42^d^ (1.34–1.50)
**Annual household income, $**			
<50,000	1 [Reference]	1 [Reference]	1 [Reference]
≥50,000	1.04^e^ (1.01–1.08)	1.04^e^ (1.01–1.08)	1.03 (1.00–1.07)
**Marital status**			
Married	1 [Reference]	1 [Reference]	1 [Reference]
Unmarried	0.99 (0.93–1.05)	0.98 (0.93–1.04)	0.99 (0.93–1.05)
Divorced/widowed/separated	0.94^d^ (0.91–0.97)	0.94^d^ (0.91–0.97)	0.94^d^ (0.91–0.97)
**Diabetes**			
Yes	1.29^d^ (1.24–1.33)	1.29^d^ (1.25–1.33)	1.29^d^ (1.25–1.33)
No	1 [Reference]	1 [Reference]	1 [Reference]
**Obesity (body mass index >30.0)**			
Yes	1.02 (0.99–1.05)	1.02 (0.99–1.05)	1.01 (0.98–1.04)
No	1 [Reference]	1 [Reference]	1 [Reference]
**Cigarette use**			
Never	1 [Reference]	1 [Reference]	1 [Reference]
Former	1.15^d^ (1.11–1.19)	1.15^d^ (1.11–1.19)	1.15^d^ (1.11–1.19)
Current	0.81^d^ (0.77–0.84)	0.80^d^ (0.77–0.84)	0.79^d^ (0.76–0.83)
**Health insurance**			
Yes	1.71^d^ (1.59–1.84)	1.72^d^ (1.60–1.85)	1.76^d^ (1.63–1.89)
No	1 [Reference]	1 [Reference]	1 [Reference]
**Has a primary care provider**			
Yes	1.71^d^ (1.60–1.83)	1.70^d^ (1.59–1.82)	1.71^d^ (1.60–1.83)
No	1 [Reference]	1 [Reference]	1 [Reference]
**Time since most recent visit to primary care provider for routine checkup**			
Within last year	1 [Reference]	1 [Reference]	1 [Reference]
1 or 2 years	0.65^d^ (0.61–0.69)	0.64^d^ (0.60–0.68)	0.63^d^ (0.60–0.67)
>2 years	0.54^d^ (0.50–0.57)	0.53^d^ (0.50–0.56)	0.52^d^ (0.49–0.56)

a All values are adjusted odds ratios (95% CI) from a multivariate model that simultaneously estimated effects for all demographic, socioeconomic, health care, and cardiovascular disease factors listed in table.

b Multivariate model adjusted for years as indicator variable (result not shown for years indicator).

c Multivariate model additionally adjusted for states as indicator variable (result not shown for states indicator).

d Significant at *P* < .001; determined by 2-sided *z* test.

e Significant at *P* < .05; determined by 2-sided *z* test.

f Significant at *P* < .01; determined by 2-sided *z* test.

**Figure 3:**
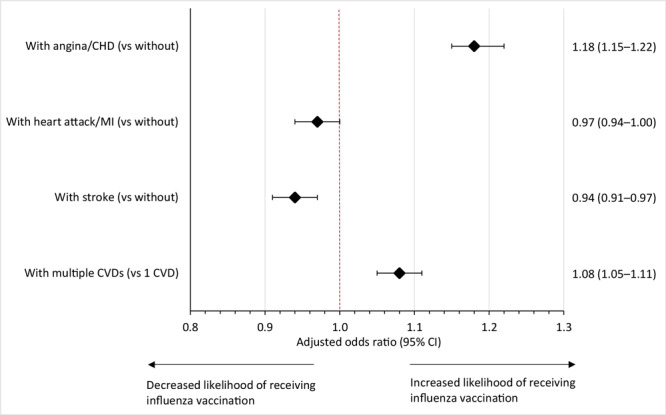
Results of multivariate regression models showing association between annual influenza vaccination and types of cardiovascular disease among US adults, Behavioral Risk Factor Surveillance System, January 2011–December 2020. Models were adjusted for reported sociodemographic characteristics, health care characteristics, and cardiovascular disease risk factors. Error bars indicate 95% CIs. Except for heart attack/myocardial infarction, odds are significant at *P* < .05 by 2-sided *z* test. Abbreviations: CHD, coronary heart disease; CVD, cardiovascular disease; MI, myocardial infarction.

The likelihood of receiving an annual influenza vaccination differed by type of CVD. The odds of receiving an annual influenza vaccination were significantly greater among adults with a history of angina/CHD (AOR, 1.18; 95% CI, 1.15−1.22; *P* < .001) than among adults without a history of angina/CHD. In contrast, odds were marginally lower among adults with a history of stroke (AOR, 0.94; 95% CI, 0.91–0.97; *P* < .001) compared with adults with no history of stroke (Figure 3).

**Table:** 

Cardiovascular disease	Adjusted odds ratio (95% CI)
**Angina or coronary heart disease**	
Yes	1.18 (1.15–1.22)
No	1 [Reference]
**Heart attack or myocardial infarction**	
Yes	0.97 (0.94–1.00)
No	1 [Reference]
**Stroke**	
Yes	0.94 (0.91–0.97)
No	1 [Reference]
**No. of cardiovascular diseases**	
≥2	1.08 (1.05–1.11)
1	1 [Reference]

## Discussion

This study found a slight improvement in influenza vaccination coverage among adults with CVD during the past decade; however, vaccination rates remained consistently below national goals (1). We found that young adults had lower vaccination rates than middle-aged and older adults, and rates among young adults did not improve during the study period. This lack of improvement may be attributed to a lower perceived risk of influenza in this population (10). The prevalence of influenza vaccination was consistently lower among middle-aged adults, supporting findings from a previous study that reported lower rates among this age group compared with adults aged 65 years or older (11). By race and ethnicity, only non-Hispanic White adults showed improvements in influenza vaccination rates. Furthermore, we found that non-Hispanic Black/African American and American Indian/Alaska Native adults were consistently less likely than non-Hispanic White adults to get annual influenza vaccinations, which may reflect persistent racial disparities in the use of preventive services and mistrust of clinical research activities (12,13). Our findings may also be attributed to various social determinants of health, including access to preventive care and treatment; such missed opportunities for preventive care and treatment among racial and ethnic minority populations merit further study (10,14).

Adults with CVD and without health insurance, without a personal health care provider, and without a recent visit to a personal health care provider for a routine checkup had lower vaccination rates than adults with health insurance, a personal health care provider, and a visit. The influence of such modifiable social determinants of health on vaccination rates highlights the underlying structural barriers, such as access to routine care, to adherence to preventive health guidelines (14). Moreover, the popularity of nonmedical settings such as workplaces, supermarkets, and drug stores as vaccination sites provides an opportunity to extend vaccination efforts beyond traditional medical settings to achieve the Healthy People 2030 target for influenza vaccination.

In this study, among adults with CVD, we found a consistently lower prevalence of influenza vaccination among current smokers than among never and former smokers. Current smoking was also identified in regression analyses as significantly lowering the odds of influenza vaccination. In contrast, among adults with CVD, former smokers (compared with never smokers) and adults with diabetes (compared with adults without diabetes) had a greater likelihood of influenza vaccination, consistent with previous literature on the general population (15,16). Smoking has contributed to nearly 25% of hospitalizations attributable to influenza, which could be prevented with vaccination (17).

In 2020, 44.3% of adults with CVD received an influenza vaccination in the US, and more than half of states are above this national average, which was the highest in any year during the study period. This relatively high prevalence was likely due to the surge in influenza vaccination uptake as protection against COVID-19 (18). During the past decade, influenza vaccination rates among adults with CVD varied significantly by state, and all states fell below the national target of 70%. Rates were comparatively higher in New England and the West North Central region and lower in the East South Central and Pacific regions. State-level differences may have been driven by preexisting social determinants of health such as economic burden, lack of transportation, lower rate of insurance coverage, vaccination mandates for certain populations, and allowed exemptions (15,19–23). Also, the discrepancy between state vaccination rates and the national goal underscores the need to further analyze data to understand the needs of states according to the unique demographic characteristics of each state. Future efforts should focus on identifying both personal and system-level barriers to uptake of influenza vaccination, including issues related to individual perceptions, resource allocation, and the infrastructure for delivering preventive care (22,24).

Our findings have important implications for state and national COVID-19 vaccination goals. The current administration has taken an active role in administering and distributing COVID-19 vaccinations. However, rollout responsibilities have still largely been borne by states, and our findings demonstrate that much work must be done to address the issue of vaccination acceptance among diverse population groups, especially among racial and ethnic minority populations, people with low socioeconomic status, people who lack health insurance, and people with comorbidities.

### Strengths and limitations

The primary strength of our study is that, to our knowledge, it is the largest and most current survey to report the national prevalence of influenza vaccination with validated survey questions on vaccination receipt (25). Moreover, the BRFSS methodology has been used and evaluated by CDC and participating states for more than 4 decades (8). In addition, our study is the first to report age-adjusted trends, by state, among adults with CVD with various sociodemographic and health care characteristics. Nonetheless, the strength of association in our findings should be interpreted with caution. The large study sample size may render weak associations significant. Furthermore, the cross-sectional design of the survey precludes causal inferences. In addition, the telephonic survey data are self-reported, so recall bias and some misclassification cannot be ruled out. However, previous studies showed that self-reported BRFSS data on influenza vaccination status and chronic conditions had better validity than self-reported data from other surveys (26–28). Although BRFSS has been conducted in all 50 states, New Jersey was not included in the 2019 survey year; furthermore, among US territories, only Guam and Puerto Rico collected data for all years, and the Virgin Islands collected data for the 2016 survey year only. We noted an approximate 6% decrease from 2017 to 2018 and then an 8 percentage-point increase in influenza coverage in 2019, similar to findings from a CDC report on vaccination coverage (29). Although the reason for the decrease in 2018 is not clear, the estimates in 2019 were consistent with other national surveillance data on influenza vaccination as reported by CDC (29,30). Also, we were not able to evaluate reasons for state-specific differences in influenza vaccination prevalence, and the reasons for opting in or opting out of vaccination. Although the COVID-19 pandemic caused disruptions in data collection for many national surveys, BRFSS was unlikely to be affected because of its use of state-of-the-art telephonic data collection methods; the response rate was 47.9% in 2020.

### Conclusion

These findings highlight significant disparities in influenza vaccination rates among adults with CVD and underline the relevance of social determinants of health toward achieving target vaccination rates (2), particularly among young people, racial and ethnic minority populations, people with comorbidities, and people who lack health insurance and a regular source of care. Our results have implications for policies on vaccine-preventable diseases, such as COVID-19, which should prioritize socially vulnerable populations and look beyond clinical settings as a place of vaccination to achieve Healthy People 2030 goals.

## Figures and Tables

**Appendix:**
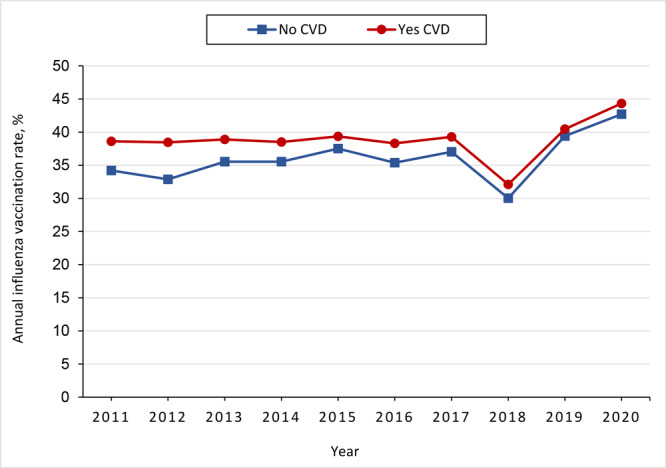
Supplementary Figure. Influenza vaccination rates among US adults, by cardiovascular disease status, Behavioral Risk Factor Surveillance System, January 2011–December 2020.

**Table:** 

CVD status	Year									
	2011	2012	2013	2014	2015	2016	2017	2018	2019	2020
No CVD, %	34.2	32.9	35.5	35.5	37.5	35.4	37.0	30.0	39.4	42.7
Yes CVD, %	38.6	38.5	38.9	38.5	39.4	38.3	39.3	32.1	40.4	44.3

## Appendix Supplemental Tables and Figure

**Appendix:** Supplemental Table 1. Characteristics of US Adults, by Cardiovascular Disease Status, Behavioral Risk Factor Surveillance System, January 2011–December 2020^a^

Characteristic	No CVD		CVD		Total	
	Unweighted no.	Weighted %	Unweighted no.	Weighted %	Unweighted no.	Weighted %
**All**	3,654,187	91.5	476,427	8.5	4,130,414	100.0
**Age, y**						
18–44	1,112,673	49.4	23,245	10.8	1,135,918	46.1
45–64	1,438,502	33.7	150,588	38.0	1,589,090	34.1
≥65	1,103,012	16.9	302,394	51.2	1,405,406	19.8
**Sex**						
Male	1,517,785	47.8	238,302	55.4	1,756,087	48.5
Female	2,135,465	52.2	237,772	44.6	2,373,237	51.5
**Race and ethnicity**						
American Indian/Alaska Native, non-Hispanic	58,553	1.6	8,448	1.6	67,001	1.6
Asian, non-Hispanic	66,264	4.1	3,397	1.6	69,661	3.9
Black/African American, non-Hispanic	275,094	11.3	38,207	11.7	313,301	11.3
Hispanic	296,619	16.5	25,200	11.0	321,819	16.0
Native Hawaiian/Pacific Islander, non-Hispanic	21,847	0.4	2,976	0.5	24,823	0.4
White, non-Hispanic	2,790,330	64.3	376,006	71.5	3,166,336	64.9
Other, non-Hispanic	89,646	1.8	13,642	2.1	103,288	1.8
**Education**						
Some high school or less	248,649	12.7	59,534	20.9	308,183	13.4
High school graduate	984,246	27.6	15,6517	31.1	1,140,763	27.9
Some college or technical school	1,007,445	31.3	133,671	30.0	1,141,116	31.2
College graduate	1,404,200	28.5	125,265	18.0	1,529,465	27.6
**Annual household income, $**						
<50,000	1,567,178	50.4	282,137	69.4	1,849,315	52.0
≥50,000	1,561,583	49.6	118,083	30.6	1,679,666	48.0
**Marital status**						
Married	1,954,282	51.1	220,467	51.0	2,174,749	51.1
Unmarried	722,808	30.4	42,869	12.0	765,677	28.9
Divorced/widowed/separated	956,241	18.5	211,030	37.0	1,167,271	20.0
**Health insurance**						
No	341,962	14.3	25,099	8.2	367,061	13.8
Yes	3,299,072	85.7	449,914	91.8	3,748,986	86.2
**Diabetes**						
No	3,225,377	90.3	325,384	68.3	3,550,761	88.4
Yes	423,215	9.7	149,878	31.7	573,093	11.6
**Obesity (body mass index >30.0)**						
No	2,430,686	71.2	290,509	62.0	2,721,195	70.4
Yes	1,010,597	28.8	167,847	38.0	1,178,444	29.6
**Cigarette use**						
Never	2,119,576	60.5	192,941	39.5	2,312,517	58.7
Former	979,931	23.0	197,128	40.2	1,177,059	24.5
Current	535,034	16.5	83,321	20.4	618,355	16.8
**Has a personal health care provider**						
No	609,553	23.4	31,179	9.0	640,732	22.2
Yes	3,030,944	76.6	443,358	91.0	3,474,302	77.8
**Time since most recent visit to personal health care provider for routine checkup**						
Within last year	2,666,863	69.7	410,628	85.8	3,077,491	71.1
1–2 Years	431,127	13.5	29,663	6.8	460,790	12.9
>2 Years	514,065	16.8	29,912	7.4	543,977	16.0

^a^ Proportions of adults with CVD and no CVD were significantly different for each characteristic at *P* <.001.

**Appendix:** Supplemental Table 2. Trends in Prevalence of Annual Influenza Vaccination Among US Adults With Cardiovascular Disease, Behavioral Risk Factor Surveillance System, January 2011–December 2020**^a^**

Characteristic	2011	2012	2013	2014	2015	2016	2017	2018	2019	2020
**No.**	52,122	51,197	50,500	49,612	43,563	53,147	45,522	47,791	42,880	39,893
**Cardiovascular disease**										
Any cardiovascular disease^b^	38.6	38.5	38.9	38.5	39.4	38.3	39.3	32.1	40.4	44.3
Angina/coronary heart disease only	39.1	39.3	40.0	38.1	40.3	40.1	40.4	37.2	42.4	46.9
Stroke only	38.6	36.2	38.0	39.5	38.8	36.9	39.7	31.7	40.2	42.9
Myocardial infarction only	32.8	37.7	35.8	34.5	37.1	34.8	37.0	28.2	37.1	40.1
≥2 Cardiovascular diseases	43.5	40.2	42.6	40.9	40.7	41.9	39.4	31.6	41.7	46.6
**Age, y**										
18–44	27.5	25.9	27.0	27.6	27.6	29.1	28.2	21.6	29.0	33.3
45–64	42.7	45.1	43.6	42.6	45.0	40.3	43.6	36.0	44.8	48.1
≥65	61.4	60.3	62.7	60.8	60.9	59.9	61.5	53.6	63.6	67.5
**Sex**										
Male	36.4	37.2	37.4	37.8	38.3	34.7	38.5	30.9	37.9	41.8
Female	41.1	39.9	40.5	39.3	40.6	42.4	40.2	33.5	43.0	47.2
**Race and ethnicity**										
American Indian/Alaska Native, non-Hispanic	41.4	58.3	37.4	37.3	40.9	36.3	31.8	25.3	42.5	40.3
Asian, non-Hispanic	34.6	41.4	36.3	44.1	39.0	51.5	54.5	38.3	60.1	50.4
Black/African American, non-Hispanic	36.2	32.6	36.3	36.3	39.1	37.1	35.2	31.0	39.8	43.3
Hispanic	33.9	32.7	33.5	32.4	34.7	34.2	35.2	30.5	33.2	36.8
Native Hawaiian/Pacific Islander, non-Hispanic	39.7	36.6	34.0	36.9	19.1	42.8	38.6	23.8	49.8	46.1
White, non-Hispanic	39.7	40.3	40.6	40.6	40.6	38.8	40.2	31.7	41.4	46.3
Other, non-Hispanic	36.7	40.0	44.5	37.7	33.3	29.9	46.6	32.7	36.6	40.4
**Education**										
Some high school or less	32.5	33.9	35.0	34.6	32.9	37.6	32.0	28.3	31.7	36.2
High school graduate	37.5	37.8	38.0	35.9	36.8	33.5	39.8	30.7	38.7	41.0
Some college or technical school	40.4	39.6	39.6	40.3	41.3	38.8	40.4	31.9	44.4	44.2
College graduate	46.2	44.4	45.2	45.3	49.1	47.9	46.7	39.5	46.8	59.1
**Annual household income, $**										
<50,000	36.7	38.1	38.1	37.2	37.0	37.0	37.6	29.8	40.2	41.8
≥50,000	44.5	41.3	42.8	44.4	47.1	44.7	43.0	35.9	43.4	49.7
**Marital status**										
Married	39.9	40.3	41.4	41.7	41.1	40.3	40.0	33.3	41.7	50.0
Unmarried	37.8	36.7	38.3	34.0	37.0	35.0	38.9	30.9	41.6	40.1
Divorced/widowed/separated	36.6	35.8	35.8	36.6	38.9	40.0	37.3	29.5	36.3	38.3
**Health insurance**										
No	22.7	24.3	21.4	21.3	24.2	23.0	23.7	17.2	25.6	24.5
Yes	43.0	41.6	42.8	42.1	41.7	40.5	42.0	34.2	43.2	48.2
**Diabetes**										
No	36.6	36.2	36.8	36.5	37.2	36.3	36.6	30.6	38.5	42.0
Yes	46.2	44.9	45.8	46.6	48.6	44.5	48.1	36.6	46.2	52.1
**Obesity (body mass index >30.0)**										
No	38.0	38.0	36.4	37.6	38.5	36.4	38.8	31.1	40.7	42.3
Yes	40.2	39.0	42.9	41.0	40.6	40.9	40.1	34.1	40.9	46.6
**Cigarette use**										
Never	41.1	40.0	38.9	39.4	40.4	39.8	40.6	33.0	43.4	45.9
Former	41.5	42.7	42.5	44.2	43.1	41.0	42.0	35.5	43.8	48.7
Current	32.1	31.6	34.9	32.3	33.4	32.1	33.8	25.8	31.9	35.9
**Has a personal health care provider**										
No	23.5	21.8	23.0	22.6	24.4	20.2	22.1	17.9	23.1	26.1
Yes	42.1	41.3	42.5	42.5	42.0	42.2	43.1	34.8	44.3	47.9
**Time since most recent visit to personal health care provider for routine checkup**										
Within last year	42.7	43.3	43.4	42.6	44.0	42.3	44.4	35.0	44.5	48.8
1–2 Years	30.7	27.7	30.4	33.0	30.5	28.0	29.4	21.1	25.3	29.4
>2 Years	25.8	23.8	24.3	22.6	23.6	24.9	21.4	14.1	17.6	20.1

^a^ All estimates were age-standardized based on the 2010 US Census population, by reported age groups. All percentages were weighted.

^b^ Any cardiovascular disease was defined as a history of stroke, myocardial infarction, coronary heart disease, or angina. Unweighted total number of cases of cardiovascular disease = 476,227.
