# Erratum, Vol. 19, October 27 Release

**DOI:** 10.5888/pcd19.220154e

**Published:** 2022-11-17

Because of a technical error, several lines of text were omitted in the online version of the first paragraph of the Results section of the article “Disparities in Influenza Vaccination Coverage and Associated Factors Among Adults with Cardiovascular Disease, United States, 2011–2020.” The missing text was added, and the corrected article was posted on October 28, 2022: https://www.cdc.gov/pcd/issues/2022/22_0154.htm. We regret any confusion this error may have caused.

